# The Formation of Social Conventions in Real-Time Environments

**DOI:** 10.1371/journal.pone.0151670

**Published:** 2016-03-22

**Authors:** Robert X. D. Hawkins, Robert L. Goldstone

**Affiliations:** 1 Department of Psychology, Stanford University, Palo Alto, CA, United States of America; 2 Department of Psychological and Brain Sciences, Indiana University, Bloomington, IN, United States of America; University of Waterloo, CANADA

## Abstract

Why are some behaviors governed by strong social conventions while others are not? We experimentally investigate two factors contributing to the formation of conventions in a game of impure coordination: the continuity of interaction within each round of play (simultaneous vs. real-time) and the stakes of the interaction (high vs. low differences between payoffs). To maximize efficiency and fairness in this game, players must coordinate on one of two equally advantageous equilibria. In agreement with other studies manipulating continuity of interaction, we find that players who were allowed to interact continuously within rounds achieved outcomes with greater efficiency and fairness than players who were forced to make simultaneous decisions. However, the stability of equilibria in the real-time condition varied systematically and dramatically with stakes: players converged on more stable patterns of behavior when stakes are high. To account for this result, we present a novel analysis of the dynamics of continuous interaction and signaling within rounds. We discuss this previously unconsidered interaction between within-trial and across-trial dynamics as a form of social canalization. When stakes are low in a real-time environment, players can satisfactorily coordinate ‘on the fly’, but when stakes are high there is increased pressure to establish and adhere to shared expectations that persist across rounds.

## Introduction

Many everyday activities are governed by strong societal conventions: the side of the road we drive on, the meaning of “red” and “green” on traffic lights, the currency we use to pay for our coffee, and the way we greet the cashier [1–4]. These are all *self-sustaining*, in the sense that we will continue to conform to the convention as long as we expect others to, and also *arbitrary*, in the sense that at least one alternative regularity exists and would be equally acceptable as long as everyone coordinated on it [5].

Equally interesting, however, are the many activities not governed by conventions. Consider a pedestrian deciding what path to take through a busy marketplace. There is still a coordination problem to solve—we do not want to keep running into one another—but we solve it ‘on the fly’ every time and there is no mutual expectation of conformity. The spatiotemporal patterns formed by pedestrians are driven more by reactive or subconscious factors than strategic or conventional considerations [6]. Similarly, there are no societal conventions governing who gets the last bite of food at a meal or what kind of music to put on afterward.

How do we account for which activities become conventionalized and which do not? First, note that all the above examples have a hierarchical structure: coordination must be achieved *within* a single interaction taking place in some environmental context, but we repeat the interaction many times, so behavior must also be maintained or adjusted *across* interactions. Certain properties of the environmental context, such as the time-course of interaction, may vary across different activities and might facilitate or inhibit the conventionalization process. Second, there is in some sense *more at stake* in the highly conventionalized activities than the less conventionalized activities: a failure to coordinate on lanes when driving could lead to severe injury or death, while a failure to coordinate on pedestrian paths could at most lead to some discomfort and social awkwardness.

To formalize and investigate these intuitions in a more rigorous, concrete game theoretic context, we developed a variation on the classic “Battle of the Sexes” game, which we call *Battle of the Exes*. Suppose there are two coffee shops in a town, one with better coffee than the other. Both you and your ex want to go out for coffee during your simultaneously occurring coffee breaks, but if you pick the same place and run into one another, neither of you will enjoy your break at all. In game theoretic terms, if players choose different locations, the better coffee shop gives a payoff of *a* and the worse coffee shop gives a payoff of *b*, with *a* > *b* > 0. However, if the two players choose the same location, neither player is given a reward (i.e. given a payoff of 0). The matrices for the payoff settings (*a*, *b*) used in our experiment are formally specified in S1 Fig.

This game has several interesting properties. Like “Battle of the Sexes”, there are three Nash equilibria for the one-shot version of the game: two (unfair) pure strategies where one player always gets the good coffee and the other player always gets the bad coffee, and one (inefficient) mixed strategy where players randomly pick between the two shops with an optimal probability determined by how much better the good coffee is (see S1 Text for a formal proof). In the repeated version of the game, there are two additional equilibria that are both fair and efficient, but both require more sophisticated patterns of coordination across rounds. Thus, there is an inherent tension between fairness and efficiency that can only be systematically resolved by the spontaneous emergence of coordinated (meta-)cooperative game play, or in other words, through conventionalization.

One possible convention is an *alternation* equilibrium: the two players take turns going to the better shop, so that they never run into each other and each get the good coffee equally often [7–9]. The other is a *correlated* equilibrium: the players both follow a reliable public signal, such as the random assignment of the better coffee to different locations. Suppose that the two shops are actually mobile carts that are randomly assigned to two locations each day. If each person picks a unique location and goes there day after day, then in the long run, the random assignment process will give them the good coffee equally often [10, 11]. Note that both fit the traditional definition of a convention according to Lewis [5], since neither convention is inherently preferable to the other, and neither player benefits from changing their behavior once the convention is established.

In this paper, we experimentally manipulate the stakes and the time continuity of the environmental context in the *Battle of the Exes* and observe the effect of each on the conventionalization process. To manipulate stakes, we simply vary the difference between the payoffs *d* = *a* − *b*, such that players incur a higher cost for failing to coordinate when *d* is higher. Our manipulation of environmental context requires more explanation. We must first introduce a class of repeated games for which each interaction unfolds in real-time.

Across 70 years of game theory research, many properties of games have been fruitfully manipulated: the payoff structure, the number of players, the number of rounds played, the information players are given regarding the others’ decisions, and the opportunity for players to communicate [12–14]. Until recently, however, most research has used games that unfold in discrete stages. These stages may be arranged simultaneously or sequentially, but players are typically given a fixed period of time to make their decision, which is recorded at the end of the period.

For many real-world decision-making tasks, there are no such timing restrictions. In the stock market, for example, brokers are free to buy or sell shares at any moment in time, not just on the hour, and their decisions immediately affect the payoffs received by the rest of the market. Thus, the trading industry, as well as a range of other economic, ecological, and social situations noted by Oprea et al [15], feature *real-time* (or *continuous*) decision-making environments, as opposed to traditional *staged* environments.

The distinction between these different decision-making environments is not only useful for appropriately formulating the above scenarios, but has been shown to critically affect outcomes. Friedman and Oprea created a real-time version of the prisoner’s dilemma game, in which players could toggle between ‘cooperate’ and ‘defect’ options at any point in time [16]. As soon as either player changed their action, the continuous flow of payoffs adjusted to a new level, which was maintained until the next change of action occurs. Total payoffs were computed by integrating these payoff curves over time. Median cooperation rates in the real-time environment increased to 90%, as opposed to 50% for the same game in an environment with 8 short discrete stages spanning the same total time as the real-time game, and close to 0% for the one-shot version. When the time period was split into finer grids of discrete stages (e.g. 60 discrete games, with each lasting 1 second), cooperation rates linearly approached those found in real-time games. Additionally, theoretical models in biology have demonstrated that when agents can respond to each other in real-time, a cooperative population evolves more easily and robustly than when interactions are restricted to discrete stages [17]. Again, this benefit increases on a continuum as the delay between stages decreases to zero.

**Fig 1:**
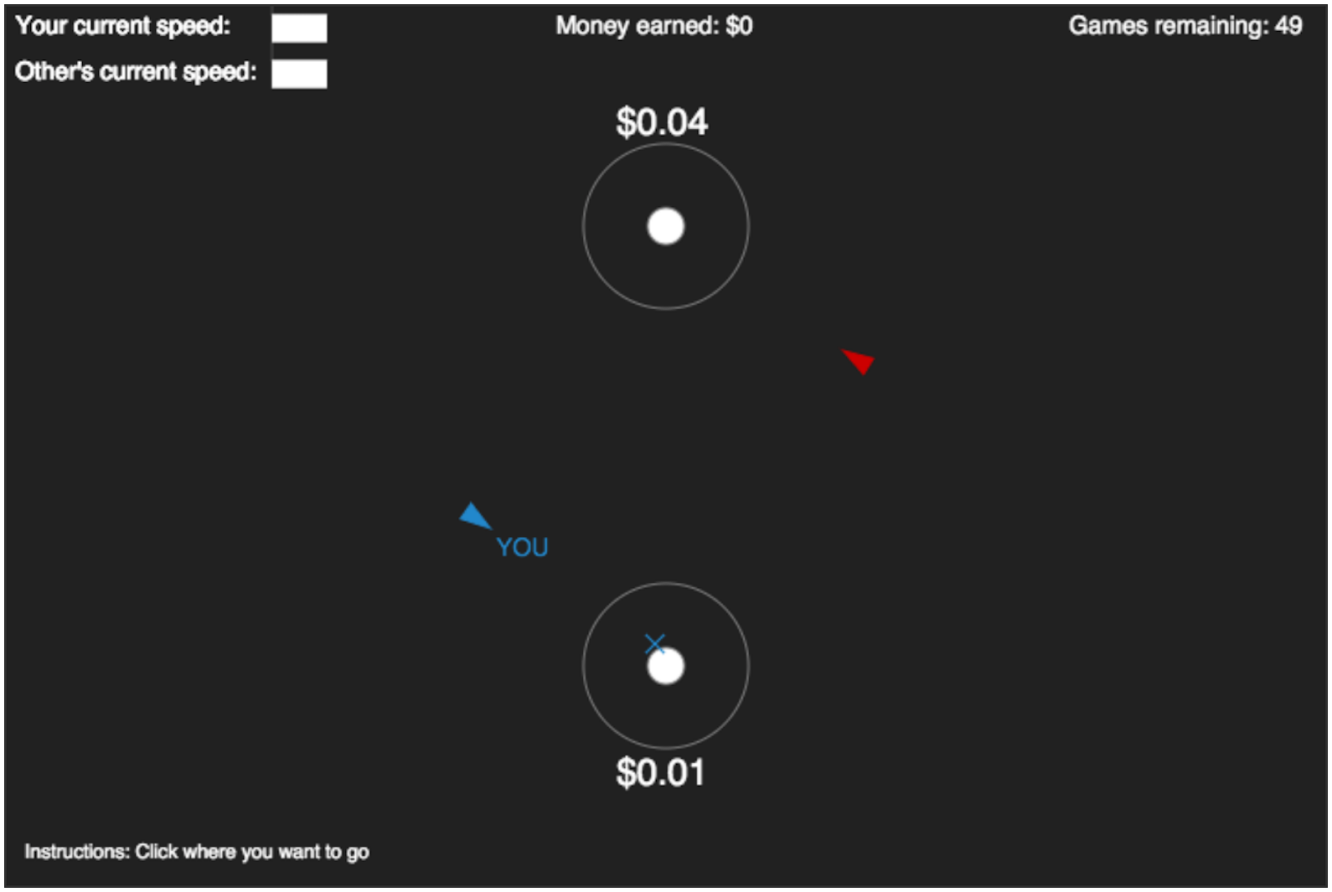
Screenshot of the first round of the experiment. The colored triangles represent the players’ avatars, the white circles represent targets (labeled by their corresponding payoffs), the outer shell gives the ‘tie radius’, and the colored cross shows a player their current ‘destination’.

These results suggest that introducing real-time interactions into a game of (impure) coordination such as the *Battle of the Exes* may substantially change the conventionalization process, although it is not clear *a priori* how it would interact with stakes and whether it would help or hinder. Many real-world activities often used as examples of convention formation—driving, walking, greeting one another—take place in real-time environments, yet empirical studies of conventionalization have exclusively relied on traditional, discrete-time environments. We directly compare these two environments in our task by using a navigational interface: each player controls an avatar, and earns payoffs by navigating their avatar to one of two target locations (see Fig 1). In the *dynamic*, real-time condition, players move at a nearly continuous pace and may change their heading at any point in time. In the *ballistic*, discrete-time condition, players simultaneously choose which target they want to go to at the beginning of the round, then have no control as their avatars go there, thus replicating the traditional paradigm with the navigational interface.

If a game is repeatedly played across multiple rounds, and each round incorporates real-time play culminating in the awarding of a payoff, then within-round and across-round player interactions can potentially shape each other. One hypothesis is that learning across rounds makes within-round coordination more efficiently achieved, as players adopt conventions learned not only from their general experience with a community [18] but also from within the immediate, local interaction [2, 19, 20]. Another hypothesis, not mutually exclusive to the first, is that within-round conflict incentivizes the creation of conventions across rounds. Players experiencing conflict in the real-time interactions of a particular round of play may strategically create coordination patterns to avoid repeating these failures to efficiently coordinate in future rounds. Both of these patterns of influence across temporal scales were explored in a novel game that rewards dyads that can coordinate their game play across trials to avoid conflict within single trials.

On a theoretical basis, we are interested in the “three levels of priority made necessary by social contract theory” proposed by game theorist Ken Binmore: efficiency, fairness, and stability [21]. Given the increased capacity for signaling and immediate reaction, we expect that the dynamic conditions of our coordination game will lead to more efficient outcomes than the ballistic conditions, regardless of the stakes. It is less obvious how a pair of players will implicitly negotiate the within-round unfairness induced by the asymmetric payoff structure, or how our two manipulations will affect the stability of the conventionalization process.

## Materials and Methods

### Experimental Framework

Experiments were performed using a suite of recent web technologies centered around Node.js. A full technical account of this experimental framework, including open-source code to replicate the task, can be found in Ref. [22].

### Ethics Statement

This manuscript reports experimental data from human subjects. Written informed consent was obtained after the nature and possible consequences of the studies were explained. The research contained in this submission was approved by the Indiana University Institutional Review Board.

### Participants

We recruited 568 participants on Amazon Mechanical Turk, yielding data for 284 dyads, which were spread across the four conditions of our factorial design. We posted 200 HITs for each condition, in separate batches. Because Mechanical Turk workers wish to participate in the experiment at unpredictable rates and times, and two participants had to be present to start a game at the same time, many players accepted $0.10 for time spent waiting and left the virtual environment before being paired with another player. Hence, we collected uneven sample sizes for each condition. Because we used non-parametric statistical methods to obtain our results, these samples sizes did not pose a problem. As with all web experiments, there was nothing to prevent participants from dropping out halfway through the experiment, or simply ignoring the experiment throughout. S1 Table summarizes the number of participants who completed the full set of rounds in each condition, as well as information about how many dropped out or were excluded due to inattention.

### Procedure

Participants were assigned to one of four conditions in a 2 × 2 experiment design. The real-time (‘dynamic’) decision-making environment and the simultaneous (‘ballistic’) environment formed one dimension. The $0.01 vs. $0.02 (‘low’) payoff discrepancy and $0.01 vs. $0.04 (‘high’) payoff discrepancy formed the other dimension. If no other players were waiting to begin a game when a participant entered the environment, they were placed in a waiting room where they could click to navigate around an empty world; if another player was already in the waiting room, a new game was immediately started. In this way, players were automatically paired into dyads. To avoid penalizing participants simply because others failed to join in time, players were eligible to submit their HIT for $0.10 after completing a pre-test. Whatever amount of money players earned from payoffs in the game was rewarded in real money as a bonus. If the game terminated before completion due to one player dropping out, players were paid whatever they had earned up to that point as a bonus.

For the ‘dynamic’ condition, players were placed at opposite ends of the virtual world (approximately 120 pixels from both targets at the top and bottom), as shown in Fig 1. The display gave the instruction “Click where you want to go”, along with information on the amount of money earned so far, the number of games remaining, and the current speeds of both players, which were held constant in this experiment. One target was always assigned the value of $0.01 and the other target always assigned $0.02 (‘low’ condition) or $0.04 (‘high’ condition), but which values appeared at the top and bottom were randomly selected at the beginning of each round. Players were given a 3 second countdown during which they had the option of secretly registering an initial destination. Once the countdown was completed, players started moving toward their destination, with full freedom to change that destination at any time. Changes in angle were immediately registered and updated in real-time within both players’ displays, but movement took place in small increments of 10 pixels every 0.67 seconds. This interface was chosen in order to minimize any effects due to lag or unequal proficiencies in using movement controls, as may be found when using arrow keys or an on-screen slider to move continuously.

For the ‘ballistic’ condition, all settings were the same, except for the initial stage. Instead of a countdown, players were first asked to click on one of the two targets, with the other player’s choice hidden from them. Once both players made a valid selection of one target, no more input was registered and the avatars began moving toward the selected destination at the same rate as the dynamic condition, but without being able to change their courses. Clicks in the vicinity of a target were auto-corrected to the center of the target so that players did not accidentally miss the target. In order to bring total payoffs closer together across all ‘low’ and ‘high’ conditions, players in the ‘low’ condition played 60 rounds, while players in the ‘high’ condition only played 50 rounds. All other settings were held constant across the stakes manipulation.

The rules governing assignment of payoffs were as follows: around each target (represented by a solid white circle), there was a visible, thin outer ring. If a player reached the inner target while any pixel of the other player’s avatar was inside this outer ring, it was counted as a tie and the round ended without awarding either player a payoff. If a player reached the inner target while the other player’s avatar was *not* within the ring, they were awarded the amount of money associated with the target, movement stopped, the round ended, and the other player was automatically awarded the amount associated with the other target, regardless of their location. This mechanism prevented a single player from trying to get both payoffs. The players were notified of the amount they earned via a message in the center of the screen saying “You earned 4¢[2¢]” At each step within a round, data on the player’s locations, angles, current earnings, and payoff assignments was written to the file. Example play for the ballistic and dynamic conditions are shown in S1 and S2 Videos, respectively. The data and code used in the following analysis are available online at https://github.com/hawkrobe/socialConventions.

## Results

### Efficiency and Fairness

Our first major result concerns the efficiency and fairness of strategic interaction. Efficiency is defined as the sum *ρ*_1_ + *ρ*_2_, where *ρ*_*i*_ is player *i*’s total payoff. It quantifies the total amount of money the players were collectively able to earn. We divide by the total amount it was *possible* to earn in order to normalize all efficiency scores to the [0, 1] interval and compare across conditions with different payoff structures. If a pair of players achieves the maximum efficiency of 1, they are optimally efficient. Note that there are many different sets of outcomes that achieve the same level of efficiency, some more fair than others.

To distinguish among these different outcomes, we introduce a measure of fairness, defined as the normalized payoff ratio

$$\begin{array}{c} \text{Fairness}=\frac{\min(\rho_{1}^{\prime },\rho_{2}^{\prime })}{\max(\rho_{1}^{\prime },\rho_{2}^{\prime })} \end{array}$$

where $\rho_{i}^{\prime }$ is the number of rounds that player *i* earned the higher payoff. This normalization maps the fairness of all conditions to the same [0, 1] interval. If one player gets the higher payoff every round, this measure of fairness will be zero; if the players finish the experiment with an equal number of times of earning the high payoff, it will be one. All of our measures are defined at the level of the dyad, and this is the unit of observation used in all of the following analyses.

**Fig 2:**
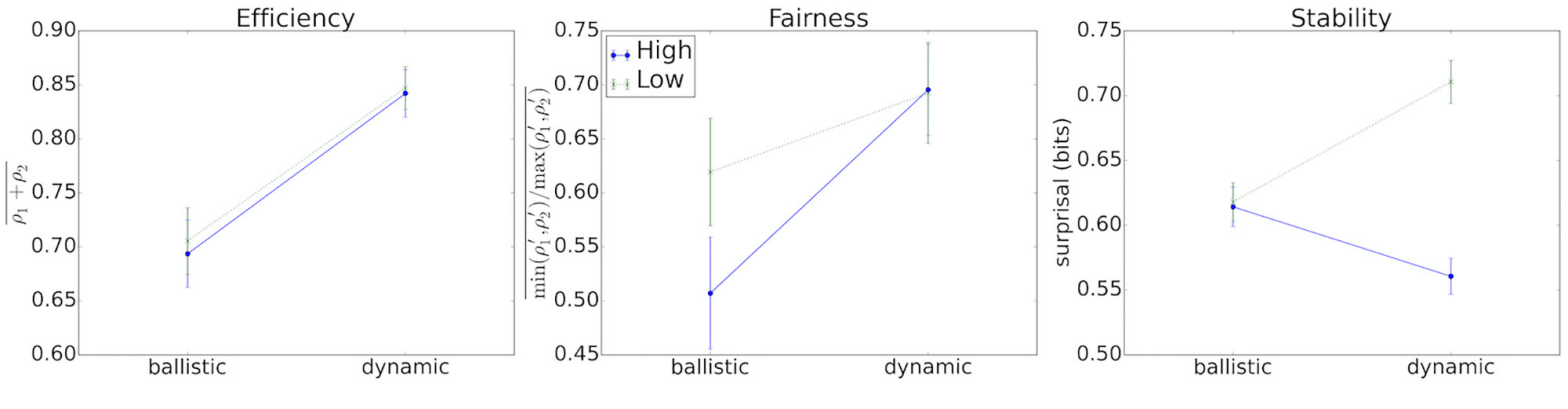
Main results at Binmore’s three levels of analysis. Bars reflect standard errors. Note that the results for the two dynamic conditions were the same by efficiency measures (a) and fairness measures (b), yet differed markedly in stability (c). Higher payoff differences increased stability (i.e. decreased surprisal) within the dynamic condition, but had no effect on stability in the ballistic condition. S3 Fig shows the same results using the less interpretable but more technically correct measure of “mean rank” on the y-axis.

Given that the four distributions of fairness scores featured varying degrees of bimodality and the efficiency scores were similarly non-Gaussian (S2 Fig), we used non-parametric techniques to test the null hypothesis of stochastic equality. For efficiency, a Kruskal-Wallis *H*-test showed a significant difference in the mean ranks of the four different distributions (*H*(3) = 30.07, *p* < .0001). Post-hoc Mann-Whitney *U*-tests at the Bonferroni corrected *α* value of .05/4 = .0125 showed that the dynamic conditions, (*M* = 0.84, *M* = 0.84), are significantly more efficient than ballistic conditions (*M* = 0.69, *M* = 0.70) both within the ‘high’ condition (*U* = 1056, *n*_1_ = 56, *n*_2_ = 69, *p* < .0001) and the ‘low’ condition (*U* = 735, *n*_1_ = 52, *n*_2_ = 46, *p* < .001). There was no main effect associated with the payoff manipulation, either at the ‘dynamic’ or ‘ballistic’ levels (*p* = .32 and *p* = .47, respectively; see Fig 2a). Given that ties are the sole mechanism through which efficiency can be lowered in the game, a more specific restatement of this result is that ties are significantly more frequent in the ballistic conditions than the dynamic conditions.

Turning to fairness, a Kruskal-Wallis *H*-test showed a significant difference in the mean ranks of the four different distributions (*H*(3) = 9.08, *p* = 0.030). Post-hoc Mann-Whitney *U*-tests at the Bonferroni corrected level of .05/4 = .0125 showed that the ‘high’ ballistic condition (*M* = .51) was significantly less fair than both the dynamic ‘high’ (*U* = 1378, *n*_1_ = 56, *n*_2_ = 69, *p* = 0.003) and 1 v. 2 (*U* = 953, *n*_1_ = 56, *n*_2_ = 46, *p* = 0.009) conditions, both of which had a mean fairness of 0.69. The fairness scores observed in the ‘low’ ballistic condition (*M* = .62) were not significantly different from the ‘low’ dynamic (*p* = 0.198) or the ‘high’ ballistic (*p* = 0.095) conditions.

### Stability

The third property in Binmore’s ‘social contract theory’ is *stability*, capturing the regularity of outcomes over multiple games, or more formally, the convergence of strategies to a point attractor. Unlike efficiency and fairness, which are summaries aggregated over all rounds, stability concerns the dynamic patterns of actions from one round to the next, thereby giving insight into how the overall efficiency and fairness was achieved. Intuitively, a strategy is stable if both players’ behavior is predictable and persistent. Knowing the outcome of a social interaction at one point in time should reduce one’s uncertainty about what will happen at other points in time. We capture this in a graded, quantitative measure using the information-theoretic measure of *surprisal*, which Shannon [23] defined as the negative logarithm of the probability of an event. In particular, we compute the average surprisal of a Markov Chain encoding the transition probabilities between events on successive rounds. An unlikely event will have low probability and an observer would therefore be highly surprised to see it happen, given knowledge of other events. This formulation is related to the model of Markov fictitious play introduced by Vanderschraaf and Skryms [7], though we only use it as a measure for data analysis rather than a cognitive model of how agents are reasoning about one another within the game (see S2 Text for technical details of the measure).

The apparent stability of a pattern of events depends upon the encoding used to define events. An obvious choice in our experiment is the outcome—who, if anyone, received the high payoff on round *t*. This can accurately capture *turn-taking* equilibria, but makes dyads who follow the *correlated* equilibrium appear highly random (though still efficient and fair). Thus, we also use a direction encoding that records whether a given player went to the top or bottom target. This encoding capture correlated equilibria, but makes turn-taking equilibria appear highly random. To be charitable, we computed stability under both encodings and recorded the more stable of the two, biasing all conditions equally toward higher stability. Note that stability is optimized in pairs that perfectly follow one of the two conventions—both conventions are point attractors in strategy space, as any divergence from the established conventions would lead to failures of coordination—and the measure gradually diverges from optimality when either (1) a convention takes longer for a pair to establish or (2) players attempt to depart from an established convention, creating ‘hiccups’ in the coded string (e.g. ABABAABA).

First, it is interesting to note that dyads in the ballistic condition have a higher prevalence of adopting a correlated equilibrium convention than those in the dynamic condition, *χ*^2^(4) = 17.18, *p* = 0.002 (see S3 Text for further details of this analysis). Second, plotting the surprisal distribution, we observe that the high-discrepancy, dynamic condition has strictly higher surprisal values than the ballistic conditions and the low-discrepancy, dynamic condition has strictly lower surprisal values (see S4 Fig). We formally checked this observation using a Kruskal-Wallis test, which showed that there are differences between the groups (*H*(3) = 98.62, *p* < .0001). Post-hoc Mann-Whitney tests at the Bonferroni corrected level of .0125 demonstrated that within the ‘low’ condition, the dynamic condition (*M* = 0.71) is significantly less stable than the ballistic condition (*M* = 0.61; *p* < .0001) and within the ‘high’ condition, the dynamic condition (*M* = 0.56) is significantly more stable than the ballistic condition (*M* = 0.62; *p* < .0001). The two dynamic conditions were significantly different from one another (*p* < .0001), but there was no significant difference between the two ballistic conditions (*p* = .06). This interaction, using surprisal as the y axis, is depicted in Fig 2c. Note that lower surprisal implies higher stability.

### Peel-off Times

Why is the high payoff difference version of the dynamic condition so much more stable than the low when they achieve the same efficiency and fairness? The answer to this question requires us to analyze the real-time dynamics within each round, which is the most unique feature of our data. When both players move toward the high payoff on a given trial, a dispute naturally arises over who should get the high payoff. Because getting the low payoff is better than a tie, one player will often ‘peel-off’ from their course toward the high payoff to concede that round’s dispute to the other player. We quantify the *degree of conflict* within a round *i* using the ‘peel-off time’ $t_{C}^{\left(i\right)}$, the percentage of round *i*’s length before one player peels off. If neither player concedes, $t_{C}^{\left(i\right)}=1$. If the players begin moving toward opposite targets at the outset of the round, $t_{C}^{\left(i\right)}=0$.

**Fig 3:**
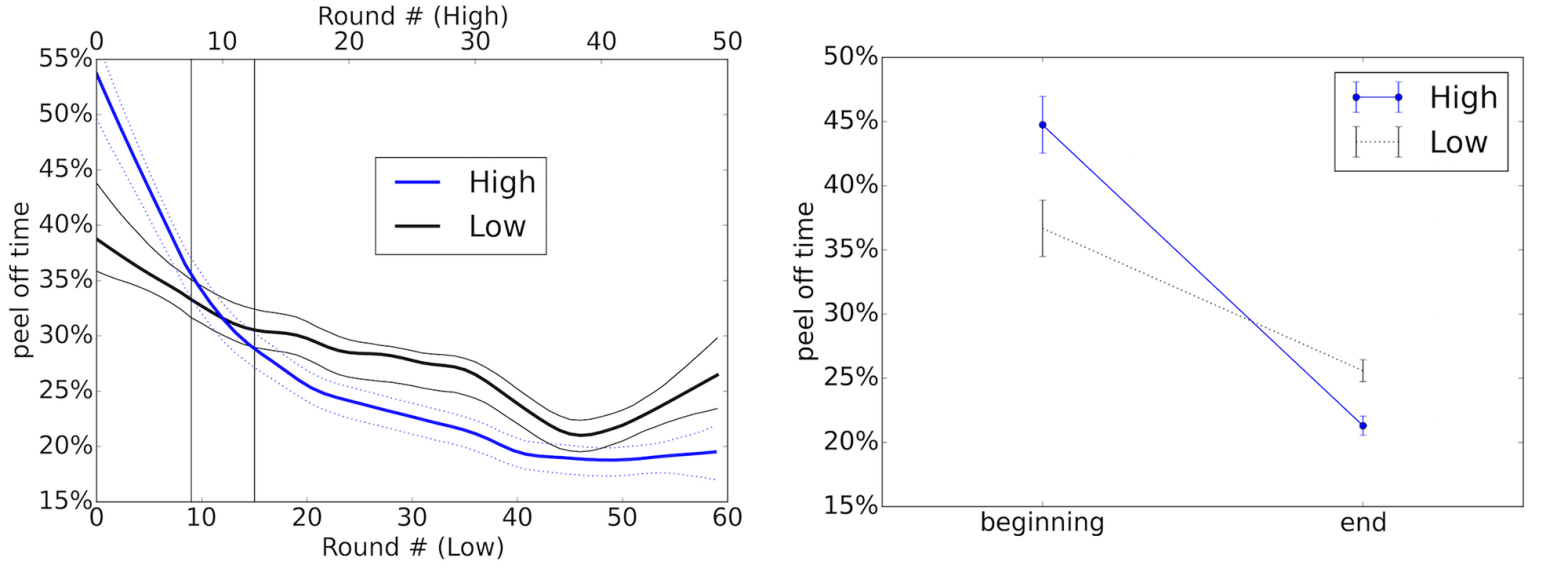
(a) Non-parametric local regression curve fitting peel-off times on each trial, broken down by ‘high’ and ‘low’ payoff conditions. Envelopes surrounding curves denote ±1 (bootstrapped) standard deviation. Note that as conventions form, we predict a decrease in peel-off times, because there is a pre-determined action for both players to take in a given round. (b) Interaction plot comparing the mean peel-off time at the beginning of the experiment against mean peel-off times at the end (with intervals indicated by the arrows in (a)). We find a crossover effect, with the ‘high’ payoff condition beginning with higher peel-off times and ending with lower peel-off times. S2 Table demonstrates the robustness of this effect to different windows.

The way peel-off time $t_{\left(i\right)}^{C}$ varies as a function of round number *i* conveys information about the formation of conventions. High peel-off times indicate a lack of consensus over which player should earn which payoff. Conventions by definition reduce this uncertainty because players conform to their prescribed actions. Regardless of which convention is being chosen (e.g. turn-taking, public signal based on location, pure dominance by one player), peel-off times are predicted to decrease as the convention is adopted. Fig 3a shows the average peel-off time for each round, smoothed using non-parametric local regression (lowess).

Note that the curve for the ‘high’ condition starts with higher peel-off times than the curve for the ‘low’ condition, and then by 1/3 of the way through the experiment has obtained lower conflict. In other words, higher initial conflict resulted in a quicker and longer-lasting adoption of conventions. Note also that the ‘low’ curve shows an uptick in conflict (i.e. a horizon effect) for the final several turns of the game, while the ‘high’ curve does not. We focus now on the first of these observations. To make the comparison rigorous, we conduct statistical tests directly on the data in the time intervals of interest, rather than smoothed curves.

We compared the peel-off times in the early interval (to the left of the first vertical line in Fig 3a) against the peel-off times in the late interval (to the right of the second vertical line). A Kruscal-Wallis test showed a significant difference in mean rank across the four groups (*H*(3) = 189.19, *p* < .0001). Pairwise Mann-Whitney tests at the Bonferroni corrected *α* level of.05/2 = .025 show that the ‘high’ condition had significantly larger peel-off times than the ‘low’ condition at early times (*U* = 71190, *n*_1_ = 378, *n*_2_ = 417, *p* = .0062) and that the ‘high’ condition had significantly smaller peel-off times at late times (*n*_1_ = 1951, *n*_2_ = 2433, *p* < .0001).

We set our interval boundaries at 20% ± 6% of the total number of rounds, where 20% is the crossover point of the curves. This is the smallest reasonable gap to place between early and late times, given that we do not expect values to differ across groups closer to the crossover point, but the result is robust across a range of larger spreads around this point (see S2 Table).

## Discussion

Our results demonstrate that while players in a real-time environment achieve higher efficiency and fairness than players in a discrete-stages environment, the stakes determined by the payoffs of individual games are crucial in determining whether a stable convention will emerge across games. We conjecture a theory of *social canalization* to explain these results, by analogy to the mechanisms of genetic assimilation and canalization in biology.

In biological evolution, genetic assimilation occurs when a phenotypic characteristic that is elicited from an environmental condition becomes genetically encoded. A classic example involves exposing drosophila embryos to ether, producing a second thorax in some proportion. If the flies are selectively bred when they produce a second thorax, by 20 generations some flies develop a second thorax even without exposure to ether [24].

A fruitful analogy can be drawn between genetic change across generations and the formation of conventions across rounds of our task, and also between the development of a single organism and the dynamics within a single round of Battle of Exes. Similar to the genetic assimilation over generations of characteristics acquired within the lifetime of an organism, conventions arise from within-round dyadic interactions over rounds of game play. The end result in both cases is that the longer temporal scale of accommodation (e.g. genetic change and dyadic convention formation) depends for its existence on the shorter temporal scale of accommodation (e.g. individual development and within-trial dynamics), but once acquired, this longer-term accommodation eliminates some of the need for the shorter temporal scale accommodation.

In our task, after many rounds of interaction, dyads interacting in real-time are typically well coordinated without any further need for inefficient within-trial dynamic interactions, particularly when the high discrepancy between payoffs places a premium on finding a coordinated solution. The greater efficiency, fairness, and stability of the (high discrepancy) dynamic condition compared to the ballistic condition indicate that the real-time dynamics within a round was instrumental for achieving these coordinating conventions across rounds.

A second interaction across temporal scales observed in biology is “canalization”, which refers to the robustness of a population’s phenotype with respect to changes in its environment and genotype [25]. The ability of organisms to learn or develop within a lifetime widens the basin of attraction surrounding an important trait, allowing a much broader range of genetic starting points to access it [26]. If there is an efficient, reliable mechanism for some trait to form during an agent’s development, there is diminished pressure to encode it in the genome.

Our game also demonstrates a social analog of biological canalization. When a dyad interacts in a dynamic, rather than ballistic, environment, then it can rely on real-time accommodation to achieve coordination and avoid ties. Experimental evidence for this comes from the greater stability of the 1 v. 2 ballistic, relative to dynamic, condition. When stakes are relatively low (i.e. there is not a large discrepancy between the payoffs), then dynamic dyads are not pressured to create an across-game social convention to assure their coordination. They can rely on within-round interaction to avoid ties, just as highly canalized organisms can rely on development within a lifetime to make up for genetic variation.

It is possible to unify these two apparently contradictory interactions across temporal scales. Sometimes, as in our high payoff discrepancy condition, real-time dynamics facilitate the creation of conventions to avoid inefficient and repetitious coordination negotiations on every round. Other times, as in our low discrepancy condition, real-time dynamics can be employed to prevent ties and promote coordination without requiring overarching conventions. Our experiment identifies *stakes* as one factor that governs whether real-time interactions will promote or obviate social convention formation. The same within-round interaction that establishes across-round conventions when the stakes are relatively high provides an acceptable low-effort alternative to establishing conventions when the stakes relatively low. Other factors that will likely affect whether the shorter temporal dynamic promotes or impedes the longer dynamical adjustment include the difficulty involved in establishing conventions, the number of rounds of iterated play, and the existing social structures for promoting coordination.

Our efficiency results are consistent with those found by Oprea and Friedman in the Prisoner’s Dilemma [16]: just as players in their real-time condition spent less time in the inefficient mutual defection profile than players in the discrete-time conditions, players in our continuous-time condition ended fewer rounds with inefficient failures of coordination. It is worth noting that our task differs from the real-time game used by Oprea and colleagues in three ways, beyond the payoff matrix. First, instead of one continuous period of game play, our dynamic condition uses a hierarchical design. Participants play many rounds, and each round takes place in real time. This more closely resembles a continuous-time version of the Hawk-Dove game used by Oprea and colleagues to test the predictions of (continuous) replicator dynamics [27], which used 20 rounds of 2-minute interactions. Second, Friedman and Oprea calculated payoffs by integrating over the whole time interval; in our task, only one payoff was given at the end of each round: payoffs were determined by the avatars’ final destinations. Third, Friedman and Oprea restricted player’s action space to a toggle between “Cooperate” and “Defect.” In our experiment, participants could in principle choose any angle at any point in time, although in practice this large space collapsed to a choice between the two target locations.

While participants in our game interacted with the same partner on every round, many real-life activities involve interacting with strangers drawn from a larger population. When we pass a car on the road, we may never have interacted with that particular driver before, but we each adhere to our mutual convention of driving on the right-hand side and successfully solve the coordination problem. How do conventions generalize to larger populations [28, 29], and how do they shift over multiple generations [30]? Our experiment cannot answer this question; we restricted ourselves to the simpler case of an isolated dyad for more precision in our manipulations. Persistent partners are more likely to produce coordination, so this is a good place to start studying the emergence of coordinated play. Additionally, it’s not clear how the conventions we studied would fare in random matching: turn-taking requires some memory of the previous round’s behavior to determine the appropriate action on the current round.

More broadly, though, the issue of generalization has been well-studied in the literature: Garrod and Doherty, for instance, explicitly manipulated whether participants in a communication game stay with the same partners or switch each round. They find that in the long-run, the latter condition creates stronger conventions [28]. This result is consistent with our theory of canalization: re-forming dyads on each round ratchets up the evolutionary pressure to establish and adhere to a social convention, similar to our 1 vs. 4 payoff condition.

The framework introduced in this paper may be fruitful in exploring other conventionalized behaviors in cognitive science. One area of particular interest is language, where some communication goals are handled ‘on the fly’ by pragmatic inference and others are encoded in the lexicon [28, 31]. The factors that determined this division and continue to shape it remain an open question. Children face the same problem in language learning; they must infer which utterances they should encode and reproduce as linguistic conventions and which should be left to pragmatics. A substantial literature in social psychology documents the cultural norms that shape our values and attitudes toward others [32], and our results suggest a high-stakes, real-time pathway through which new norms may emerge. Our analysis also contributes to the broader groundswell of interest in cognitive science [33–35], economics [15, 16], and biology [17] in exploring the trajectory of decision processes as they unfold in real time.

## Supporting Information

S1 Video Demonstration of game play in ballistic condition. (MOV)

S2 Video Demonstration of game play in dynamic condition. (MOV)

S1 Text Proof of the three Nash equilibria for the one-shot “Battle of the Exes” game. (PDF)

S2 Text Technical details for computing stability. (PDF)

S3 Text Chi-Squared Test for whether different conventions tend to emerge in different conditions. (PDF)

S1 Fig Payoff Matrix for “Battle of the Exes”. There are two coffeeshops in town, one with better coffee than the other. Both individuals would prefer to go to the coffeeshop with better coffee, but only if the other will not be there. If they run into either other, they are unhappy and get nothing.
(TIFF)

S2 Fig Empirical distribution of Efficiency, Fairness, and Stability measures for each condition. Note that our measure of fairness is not normal and does not keep the same shape across conditions, hence we must use non-parametric Kruskal-Wallis and Mann-Whitney tests to compare their stochastic ordering.
(TIF)

S3 Fig Efficiency, Fairness, and Stability results using mean rank. Fig 2 in the main text uses interpretable means on the y axis, but because the Kruskal-Wallis and Mann-Whitney tests are based on mean rank rather than the mean of the sample distribution, it is technically more correct to visualize the differences using mean rank. Note, however, that the qualitative patterns visible in Fig 2 are identical with the patterns here, so the visualization remains reliable.
(TIFF)

S4 Fig Surprisal CDFs for all conditions. Note that the ‘low’ dynamic condition lies above the other curves over the entire range of values and that the ‘high’ dynamic condition lies below the other curves.
(TIFF)

S5 Fig Surprisal analysis pipeline. Our pipeline of analysis from outcome time series (top row) to surprisal time series (middle row) to surprisal distributions (bottom row). The left column demonstrates what is intuitively a stable equilibrium, with some initial struggle converging into an alternation pattern. The right column demonstrates what is intuitively a less predictable or more unstable equilibrium, which has a much more erratic surprisal time series. The mean surprisal for the left column is consequently much smaller than the mean surprisal for the right column.
(TIFF)

S6 Fig Parameter robustness for estimating the Markov Chain and computing surprisal values. *m* determines how many steps back the Markov Chain looks when estimating the probability of transitions. The results shown in the main text are robust across many choices for this parameter.
(TIFF)

S1 Table Breakdown of dyads by condition. Dyads counted in “# included” were included in all analyses. Dyads in “# uncompleted” were excluded from the analyses because one player dropped out before completing the experiment. Note that all four conditions had roughly the same drop-out rate. Dyads in “# not paying attention” were excluded from the analyses because one or more players in the game allowed 5 or more rounds to pass without providing any input, indicating that they stopped paying attention.
(PDF)

S2 Table Robustness of peel-off time analysis to different window sizes. All Kruscal-Wallis tests are significant at the *α* = .001 level, and all post-hoc Mann-Whitney tests are significant at the Bonferroni corrected level of *α* = .05/2 = .025. Note that the ‘high’ condition has greater peel-off times for early round, but lower peel-off times for later rounds.
(PDF)
