# Supplementary material for: The Formation of Social Conventions in Real-Time Environments
Source: PLoS One. 2016 Mar 22;11(3):e0151670. doi: 10.1371/journal.pone.0151670 (PMC4803472; doi:10.1371/journal.pone.0151670)
Supplement: S1 Text — (PDF) [file pone.0151670.s003.pdf]

Derivation of Nash equilibria for one-shot "Battle of the Exes"

The payoff matrix for "Battle of the Exes" has the same properties as "Battle of the Sexes," but remapped to the off-diagonal. Like Battle of the Sexes, there are 3 Nash equilibria in the one-shot game: two pure equilibria and one mixed equilibrium. It is useful to prove this, in order to see why each are deficient in some way and can be avoided in the repeated version of the game. Let "G" denote the action of "going to the good coffeeshop" and let "N" denote to "going to the not-so-good coffeeshop". Then each player has the strategy set $S_i = \{G, N\}$. A strategy profile S for a given game is a tuple specifying a strategy from each player's strategy set: $S = S_1 \times S_2$. Then $S = (s_1, s_2) = (G, G)$ corresponds to the strategy profile where both players choose the good coffeeshop, $S = (G, N)$ corresponds to the profile where player 1 chooses the good coffeeshop and player 2 chooses the not-so-good one, and so on. Finally, let $\pi_i : S \to \mathbb{R}$ be the payoff function for player i given strategy profile S.

First, it is easy to see that $S = (G, N)$ and $S = (N, G)$ are pure Nash equilibria; if player 1 knows with certainty that player 2 will choose one coffeeshop, it is always in the first player's best interest to choose the other. Any unilateral change in strategy will result in a payoff of 0. Formally, for all players i and strategy profile $S = (\pi_i(s_1, s_2 | s_i \neq s_2) \geq \pi_i(s_1, s_2 | s_1 = s_2) = 0$. Note that this strategy is unfair: one player always gets a higher payoff than the other.

Next, we derive the mixed strategy Nash equilibrium for the more general game where the lower payoff is 1 and the higher payoff is T. Then we can plug in the values of $T = 2$

and $T = 4$ to find the specific strategies for our payoffs. In mixed strategies, players assign probabilities to different strategies. Let p be the probability that player 1 chooses action "G", then there is a probability of $1 - p$ that they choose action "N". Similarly, let q be the probability that player 2 chooses action "G". Then there is a probability of $1 - q$ that they choose action "N". In order for the players to randomize, the expected values corresponding to their two actions must be equal. This forms a system of equations that we can solve for p and q. First, we compute the expected values for player 1:

$$\mathbb{E}[\pi_1(G, s_2)] = \sum_{s_2 \in S_2} \pi_1(G, s_2) = 0 \times q + T \times (1 - q)$$

$$\mathbb{E}[\pi_1(N, s_2)] = \sum_{s_2 \in S_2} \pi_1(N, s_2) = 1 \times q + 0 \times (1 - q)$$

Setting these expected values equal to one another, we get the equation $T(1 - q) = q$. This implies that $q = \frac{T}{T+1}$. In other words, if player 2 uses these probabilities, all of player 1's strategies will have the same expected payoff. Similarly, when we compute expected values for player 2:

$$\mathbb{E}[\pi_2(s_1, G)] = \sum_{s_1 \in S_1} \pi_2(s_1, G) = 0 \times p + T \times (1 - p)$$

$$\mathbb{E}[\pi_2(s_1, N)] = \sum_{s_1 \in S_1} \pi_2(N, s_1) = 1 \times p + 0 \times (1 - p)$$

and set them equal, we get $T(1 - p) = p$, implying $p = \frac{T}{T+1}$. Thus, in this game, the mixed strategy is for each player to go to the good coffeeshop with probability $\frac{T}{T+1}$.

Because both players have chosen their mixture probabilities to make their opponent indifferent across strategies, any deviation from this mixture gives exactly the same payoffs,

making this a Nash equilibrium. Unfortunately, this combination of strategies, denoted $\sigma = (\sigma_1, \sigma_2)$ has a relatively low payoff for each player:

$$
\begin{aligned}
\pi_1(\sigma_1, \sigma_2) &= P(G, G|\sigma) \times 0 + P(B, G|\sigma) \times 1 + P(G, B|\sigma) \times T + P(B, B|\sigma) \times 0 \\
&= pq \times 0 + (1-p)q \times 1 + p(1-q) \times T + qq \times 0 \\
&= \tfrac{T}{(T+1)^2} + \tfrac{T^2}{(T+1)^2} \\
&= \tfrac{T}{T+1}
\end{aligned}
$$

and similarly for player 2, since the game is symmetric. For $T = 2$, each player goes to the good coffeeshop with probability 2/3, yielding an expected payoff of 2/3. For $T = 4$, each player goes to the good coffeeshop with probability 4/5, yielding an expected payoff of 4/5. Note that even as $T \to \infty$, the expected payoff is bounded by 1, which is the amount they could get by playing the pure equilibrium where they get the lower payoff of 1. In this sense, the mixed equilibrium is highly inefficient. However, it does have the property of being fair, since both players have the same expectation. The repeated version of the game, of course, gives rise to a much larger space of strategies, which we do not attempt to enumerate or classify, choosing instead to focus on the alternation and public signal strategies found empirically.
