# Supplementary material for: The Formation of Social Conventions in Real-Time Environments
Source: PLoS One. 2016 Mar 22;11(3):e0151670. doi: 10.1371/journal.pone.0151670 (PMC4803472; doi:10.1371/journal.pone.0151670)
Supplement: S2 Text — (PDF) [file pone.0151670.s004.pdf]

Computing stability

Consider a single pair of players' time series of game outcomes, which can be encoded as a sequence of states S. Each $s \in S$ is drawn from the alphabet $\Sigma = \{R, B, T\}$, with R indicating 'red wins', B indicating 'blue wins' and T indicating 'tie.' This is an exhaustive set of outcomes. Let S_m be the set of all subsequences of length m. We train a Markov Chain of order m on this sequence of states such that

$$\widehat{P}(X_t | X_{t-1}, \dots X_{t-m}) = \frac{\sum_{s \in S_m} \mathbb{I}_{\{s = X_{t-m} \cdots X_{t-1} X_t\}} + 1/3}{\sum_{s \in S_{m-1}} \mathbb{I}_{\{s = X_{t-m} \cdots X_{t-1} + 1\}}}$$

where $\mathbb{I}_b$ is the indicator function returning 1 when b is true. Note that by adding 'virtual counts' for each state in the alphabet, we are using the Bayesian maximum a priori (MAP) estimator with a uniform prior over each conditional distribution. This prevents us from overreliance on too little data. Note that as the sample size grows, the virtual counts will contribute less and less to the estimate, such that this estimator converges to the maximum likelihood estimator [1].

Once we learn these conditional probabilities, we can compute Shannon's (conditional) surprisal

$$S(t) = -\log_2[P(x_t | x_{t-1}, \dots, x_{t-m})]$$

for each step t in the time series, where x_t is the outcome and $P(x_t | x_{t-1}, \dots, x_{t-m})$ is the probability of that outcome, according to the Markov chain [2]. This creates a second

time series consisting of surprisals for each outcome given the entire time course of coupled dynamics. Note that a stable equilibrium shows up as a long period of low surprisal in this time series.

Following this observation, we define the *stability* for a particular pair of participants as the distribution of surprisals their outcomes generate (see S5 Fig. for examples of each step in this process). To compare conditions at the group level, we aggregated the total set of surprisals computed for a condition into one long list (e.g. for the high-discrepancy, dynamic condition, there are 69 pairs and $50 - m$ surprisal values for each pair, so for an order 2 Markov chain, the condition as a whole has 3312 surprisal values), and take the mean. The resulting stability for each condition was consistent across different choices of m, the 'memory' of the process (see S6 Fig.) See S4 Fig. for a qualitative comparison of the surprisal CDFs across all conditions.

References

[1] Young GA, Smith RL. Essentials of statistical inference. vol. 16. Cambridge University Press; 2005.

[2] Shannon CE. A mathematical theory of communication. Bell system technical journal. 1948;27.
