# Supplementary material for: The Formation of Social Conventions in Real-Time Environments
Source: PLoS One. 2016 Mar 22;11(3):e0151670. doi: 10.1371/journal.pone.0151670 (PMC4803472; doi:10.1371/journal.pone.0151670)
Supplement: S3 Text — (PDF) [file pone.0151670.s005.pdf]

Analysis of different conventions in different conditions

For each dyad, we determined whether the 'direction' encoding or the 'outcome' encoding was more stable. The 'direction' encoding is highly stable for a public signal equilibrium based on the random assignment of high and low targets, while the 'outcome' encoding is highly stable under a turn-taking equilibrium. For each condition, we counted the number of dyads falling into each class, yielding a 2x2x2 contingency table. We conducted a χ^2 contingency test on this table to check whether the true frequencies differed from those expected if stability were independent of the condition.

We found a significant relationship between the condition and which encoding is the best fit ($\chi^2(4) = 17.18, p = .002$). In particular, the frequency of 'outcome' encodings (corresponding to 'turn-taking' equilibria) is greater than expected in the 'high' dynamic condition, and the frequency of 'direction' encodings (corresponding to 'correlated' equilibrium) is greater than expected in the 'low' dynamic condition. This indicates in general that the real-time environment favors turn-taking conventions and the discrete-stage environment favors a coordinating signal convention. Game theory cannot currently account for why different environments favor different equilibria (given that the Nash solution concept does not rank multiple equilibria), making this an interesting phenomenon to pursue in follow-up studies.
