# Supplementary figures and images for: The Formation of Social Conventions in Real-Time Environments
Source: PLoS One. 2016 Mar 22;11(3):e0151670. doi: 10.1371/journal.pone.0151670 (PMC4803472; doi:10.1371/journal.pone.0151670)

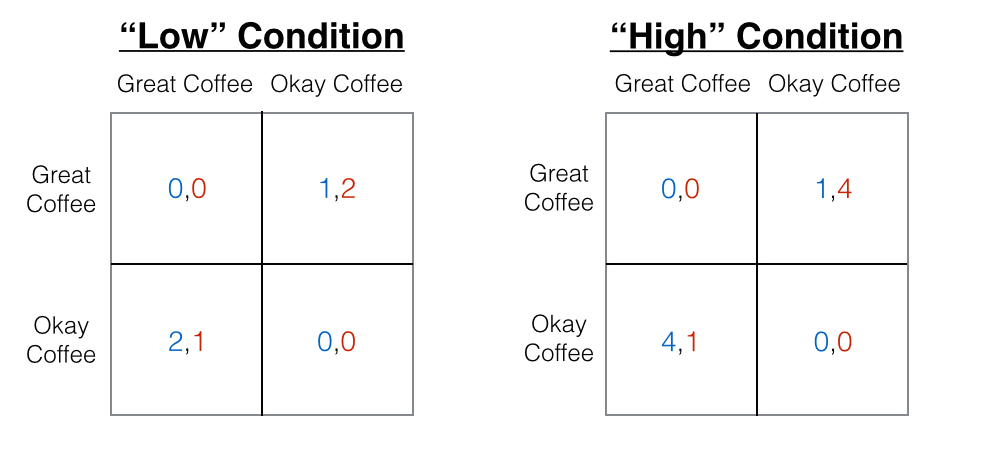

Supplement: S1 Fig — There are two coffeeshops in town, one with better coffee than the other. Both individuals would prefer to go to the coffeeshop with better coffee, but only if the other will not be there. If they run into either other, they are unhappy and get nothing. (TIFF) [file pone.0151670.s006.tiff]

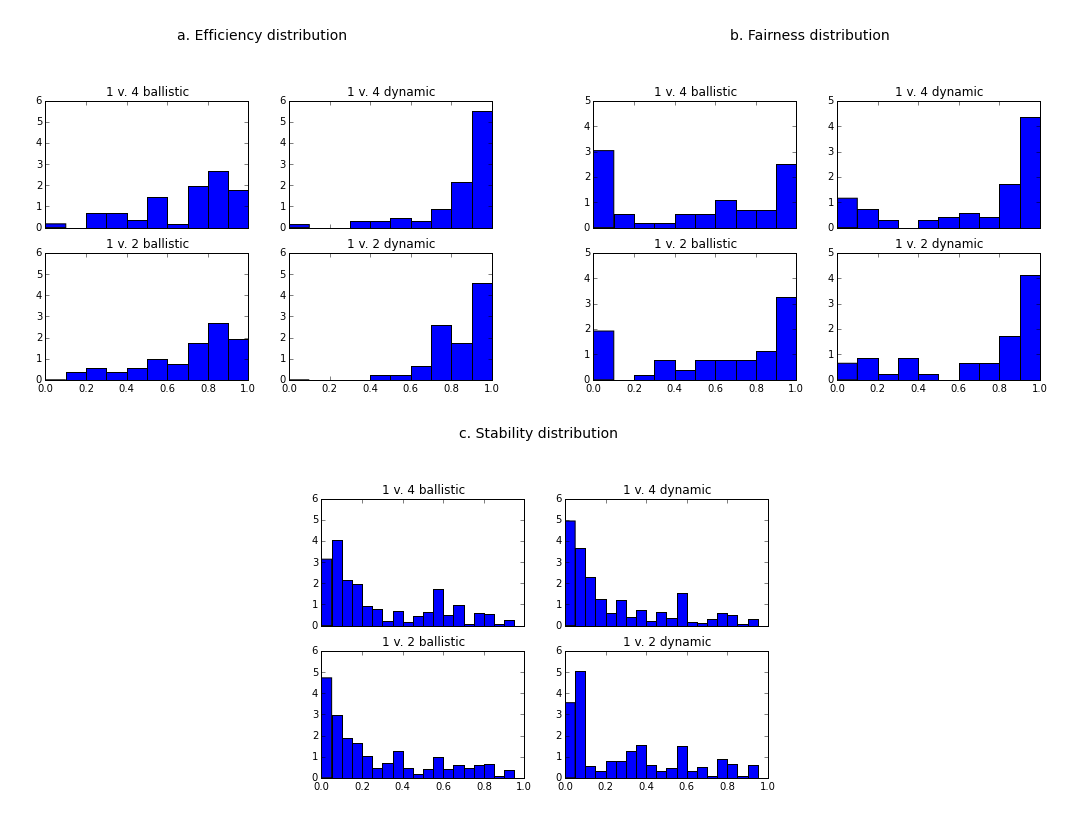

Supplement: S2 Fig — Note that our measure of fairness is not normal and does not keep the same shape across conditions, hence we must use non-parametric Kruskal-Wallis and Mann-Whitney tests to compare their stochastic ordering. (TIF) [file pone.0151670.s007.tif]

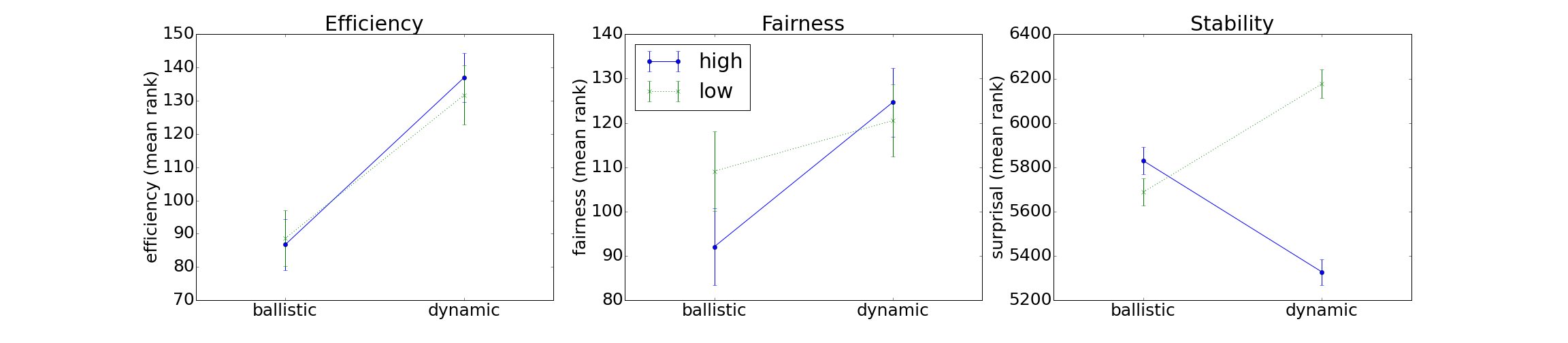

Supplement: S3 Fig — Fig 2 in the main text uses interpretable means on the y axis, but because the Kruskal-Wallis and Mann-Whitney tests are based on mean rank rather than the mean of the sample distribution, it is technically more correct to visualize the differences using mean rank. Note, however, that the qualitative patterns visible in Fig 2 are identical with the patterns here, so the visualization remains reliable. (TIFF) [file pone.0151670.s008.tiff]

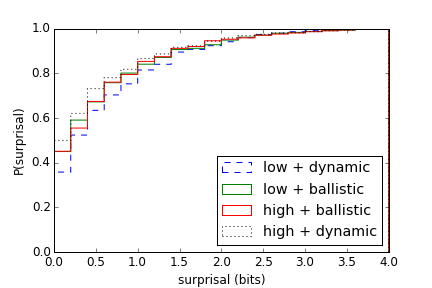

Supplement: S4 Fig — Note that the ‘low’ dynamic condition lies above the other curves over the entire range of values and that the ‘high’ dynamic condition lies below the other curves. (TIFF) [file pone.0151670.s009.tiff]

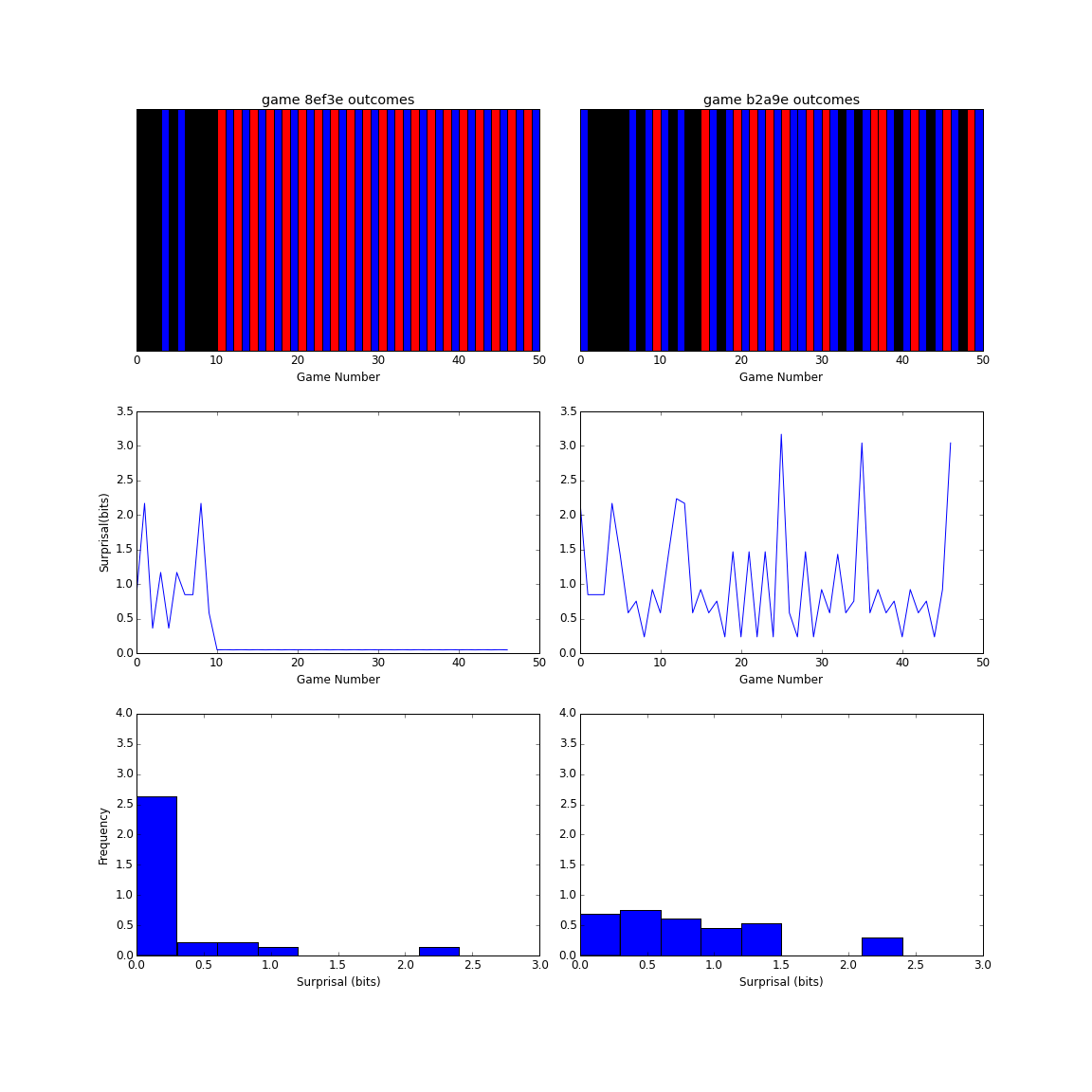

Supplement: S5 Fig — Our pipeline of analysis from outcome time series (top row) to surprisal time series (middle row) to surprisal distributions (bottom row). The left column demonstrates what is intuitively a stable equilibrium, with some initial struggle converging into an alternation pattern. The right column demonstrates what is intuitively a less predictable or more unstable equilibrium, which has a much more erratic surprisal time series. The mean surprisal for the left column is consequently much smaller than the mean surprisal for the right column. (TIFF) [file pone.0151670.s010.tiff]

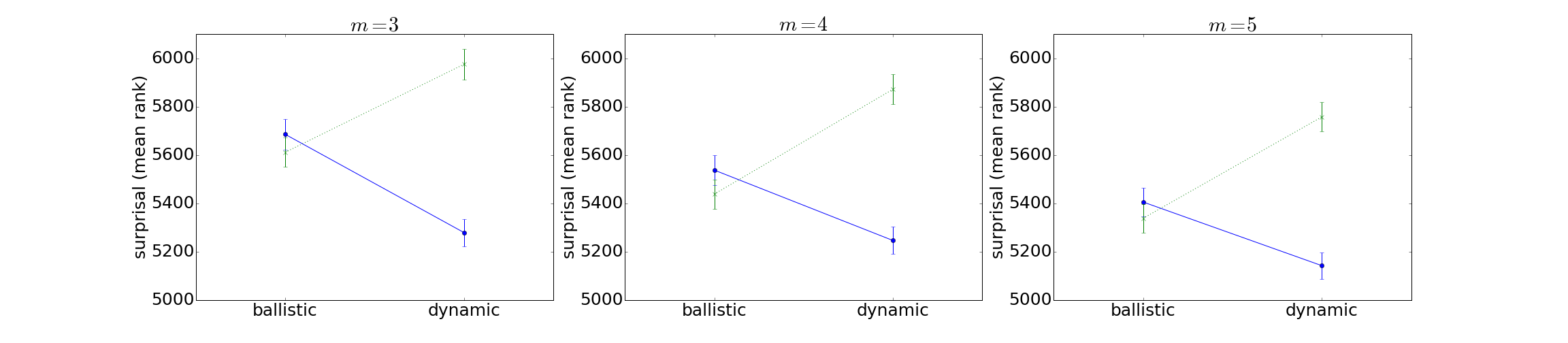

Supplement: S6 Fig — m determines how many steps back the Markov Chain looks when estimating the probability of transitions. The results shown in the main text are robust across many choices for this parameter. (TIFF) [file pone.0151670.s011.tiff]
