# Supplementary material for: The Formation of Social Conventions in Real-Time Environments
Source: PLoS One. 2016 Mar 22;11(3):e0151670. doi: 10.1371/journal.pone.0151670 (PMC4803472; doi:10.1371/journal.pone.0151670)
Supplement: S1 Table — Dyads counted in “# included” were included in all analyses. Dyads in “# uncompleted” were excluded from the analyses because one player dropped out before completing the experiment. Note that all four conditions had roughly the same drop-out rate. Dyads in “# not paying attention” were excluded from the analyses because one or more players in the game allowed 5 or more rounds to pass without providing any input, indicating that they stopped paying attention. (PDF) [file pone.0151670.s012.pdf]

Condition		# included	# uncompleted	# not paying attention
High	Dynamic	69	16	8
	Ballistic	56	9	–
Low	Dynamic	46	14	4
	Ballistic	52	10	–
