# Supplementary material for: The Formation of Social Conventions in Real-Time Environments
Source: PLoS One. 2016 Mar 22;11(3):e0151670. doi: 10.1371/journal.pone.0151670 (PMC4803472; doi:10.1371/journal.pone.0151670)
Supplement: S2 Table — All Kruscal-Wallis tests are significant at the α = .001 level, and all post-hoc Mann-Whitney tests are significant at the Bonferroni corrected level of α = .05/2 = .025. Note that the ‘high’ condition has greater peel-off times for early round, but lower peel-off times for later rounds. (PDF) [file pone.0151670.s013.pdf]

		Early peel-off times			Late peel-off times		
Interval Range	Kruscal-Wallis P	L mean	H mean	P value	L mean	H mean	P value
0.2 ± 0.06	$< .001$	0.37	0.45	0.006	0.26	0.21	$< .001$
0.2 ± 0.08	$< .001$	0.38	0.46	0.008	0.25	0.21	$< .001$
0.2 ± 0.10	$< .001$	0.40	0.48	0.023	0.25	0.21	$< .001$
0.2 ± 0.12	$< .001$	0.41	0.49	0.017	0.25	0.21	$< .001$
0.2 ± 0.14	$< .001$	0.40	0.53	0.003	0.25	0.21	$< .001$
0.2 ± 0.16	$< .001$	0.40	0.55	0.005	0.25	0.21	$< .001$
